# The Impact of Onset Age on Eosinophils in Kawasaki Disease

**DOI:** 10.3390/biomedicines10040835

**Published:** 2022-04-01

**Authors:** Yu-Jhen Chen, Mindy Ming-Huey Guo, Ling-Sai Chang, Ho-Chang Kuo

**Affiliations:** 1 Department of Pediatrics, Kaohsiung Chang Gung Memorial Hospital, Chang Gung University College of Medicine, Kaohsiung 83301, Taiwan; jun813@mail2000.com.tw (Y.-J.C.); mindymhguo@yahoo.com.tw (M.M.-H.G.); joycejohnsyoko@gmail.com (L.-S.C.); 2 Kawasaki Disease Center, Kaohsiung Chang Gung Memorial Hospital, Kaohsiung 83301, Taiwan; 3 College of Medicine, Chang Gung University, Taoyuan 33302, Taiwan

**Keywords:** age, eosinophil, Kawasaki disease, Z-score, coronary artery lesion, allergy, IVIG

## Abstract

(1) Background: Kawasaki disease (KD) mainly affects children under the age of 5 years and eosinophilia in KD patients might be associated with the development of allergic diseases. We compared the age-adjusted Z-score (Z) of eosinophils and aimed to evaluate the impact of onset age on eosinophils in KD patients. (2) Methods: We divided 398 KD patients into seven age subgroups. Laboratory data and the age-adjusted Z-score of eosinophils during the phases of Kawasaki disease were analyzed. (3) Results: The absolute eosinophil count among all age groups showed significant differences in the post-intravenous immunoglobulin (IVIG) phase and throughout the course of KD with Z-score adjusted for age. Further analysis showed persistent elevation of the age-adjusted Z-score of eosinophils (Z-eosinophil) especially in the under six-month-old age subgroup. In addition, we divided the Z-eosinophil into two groups to find the relationship with coronary artery lesions (CALs). Patients with a higher eosinophil count than average age values had a higher risk of developing CALs, while those with a lower eosinophil count than average age values had a lower risk of having CALs. (4) Conclusions: These findings may provide information to clinicians to pay attention to allergic diseases during the follow-up of KD, especially for children who are younger than 6 months old at the onset of KD, and eosinophil count could be a crucial focus in KD.

## 1. Introduction

Kawasaki disease (KD) is a systemic vasculitis of unknown etiology that mainly affects children under the age of 5 years [1]. The most common complication of KD is coronary artery lesions (CALs), therefore, KD is the leading cause of acquired heart disease in children [1]. Previous studies have shown that eosinophilia could be a powerful factor for differentiating KD from febrile children, as well as may be related to CALs and intravenous immunoglobulin (IVIG) resistance [2,3,4,5]. Furthermore, we previously reported that the T helper type 2 (Th2) immune response was elevated in the acute phase of KD [6]. Therefore, eosinophils may be potentially linked with allergic diseases in KD patients [4,6,7]. However, the baseline normal range of eosinophil levels varies by age groups [8,9,10]. Differing incidence and immunological variability according to age may contribute to the differential pathophysiology of KD. Therefore, we used the Z-score to correct age bias and used data of Asian children for the standard values [8]. In this study, we aimed to evaluate the impact of onset age on eosinophils and provide more evidence establishing the importance of eosinophils in KD.

## 2. Materials and Methods

We enrolled 398 KD patients who met the American Heart Association (AHA) criteria during the period from 2005 to 2018 at the Kaohsiung Chang Gung Memorial Hospital in Taiwan. This retrospective study was reviewed and approved by the Institutional Review Board of Chang Gung Medical Foundation (201900827B0, 14 September 2020). There were 126 children who had CALs but no significant difference in the age subgroups was found (*p* = 0.57). The definition of a CAL Z-score is described by the AHA as an evaluation of coronary artery dilation severity by correcting for body surface area (BSA) [1]. A coronary artery Z-score <2 means no involvement [1]. We defined a CAL as including dilation of coronary vessels and all sizes of coronary artery aneurysms. Among the patients, 4 patients had a history of allergies, one patient had medication (a leukotriene receptor antagonist), and one patient had a family history of asthma. The average days of illness at the time of blood collection was 5.29 ± 0.14 days. The KD patients were all treated with at least a single dose of IVIG (2 g/kg infusion for 12 h), and blood samples were collected within 24 h before IVIG treatment (pre-IVIG), 3 days after completing the initial IVIG treatment (post-IVIG), and at least 3 weeks after IVIG treatment, which were considered subacute phase samples. We excluded patients who did not complete all of the treatment and who were loss to follow-up post-IVIG (*n* = 27) or in the subacute phase (*n* = 162). We prescribed low-dose Aspirin (3–5 mg kg^−1^ d^−1^) to patients without coronary artery changes and continued for another 4 to 6 weeks after onset of illness. We analyzed the absolute eosinophil count (AEC) and the age-adjusted Z-score of eosinophils (Z-eosinophil) in seven KD age subgroups during the course of Kawasaki disease. The mean values and standard deviation (SD) for various age groups had already been established for Chinese children in a previous study [8]. The Z values were calculated using the equation provided below. The Z value was corrected for age-related biases according to chronological age, and the SD was needed to perform the calculation. Finally, we were able to obtain Z-eosinophil, and then compared characteristics to the absolute eosinophil counts in each age group (Equation (1)):

(1) $$Z\;value=\left(\frac{measure\;value-mean\;value}{\mathrm{standard}\;\mathrm{deviation}}\right)$$

All statistical tests were performed using SPSS 14.0 (SPSS for Windows, version 14, SPSS, Chicago, IL, USA). We used the Kolmogorov–Smirnova test to examine the data and to reveal non-normal distributions. We carried out the Kruskal–Wallis and Mann–Whitney U tests to estimate the significance of incidence. *p*-values < 0.05 were considered to be statistically significant.

## 3. Results

### 3.1. Age-Adjusted Z-Score of Eosinophils and Absolute Eosinophil Count during KD Course

According to Yang et al., the normal range of eosinophil counts differs among age groups [8]. To further investigate which age group had the most significant eosinophil count difference during the course of KD, we divided 398 KD patients into 7 subgroups (Table 1) [8]. Eosinophilia was defined as the presence of eosinophils >350 cells/mm^3^, and the AEC could be calculated by multiplying the total white blood cell count against the percentage of eosinophil count [10]. The evaluation of the AEC during the acute phase of KD showed eosinophilia among all age subgroups, but without statistical significance. After adjusting the AEC with Z-scores adjusted for age, we found a significant difference (*p* < 0.001) among the age subgroups, as shown in Table 1. Three days after starting IVIG treatment, we recorded lab data of 371 patients in the acute phase and 236 patients in the subacute phase. The mean of the AEC decreased during the course of KD but the Z-eosinophil still showed significant differences among age subgroups, not only in the post-IVIG phase (*p* < 0.001) but also in the subacute phase (*p* < 0.001) (Table 1). We observed that the subgroup under 1 year old had the most significant difference of Z-eosinophil in both the pre-IVIG phase (2.14 ± 0.28 vs. 0.75 ± 0.13, *p* < 0.001) and post-IVIG phase (1.58 ± 0.24 vs. 0.35 ± 0.12, *p* < 0.001), as shown in Table 2. Furthermore, in the subacute phase, the KD patients younger than 6 months showed significantly higher Z-eosinophil (2.14 ± 0.67 vs. 0.40 ± 0.12, *p* < 0.001) (Table 2).

### 3.2. Age-Adjusted Z-Score of Eosinophils and Coronary Artery Lesion

**Figure 1:**
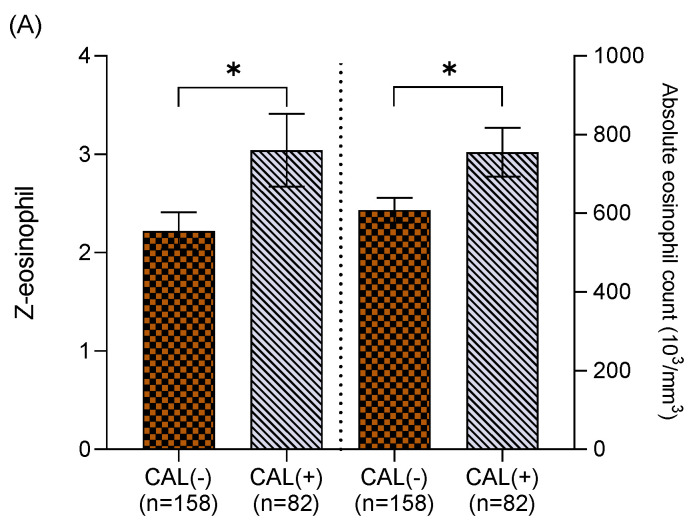

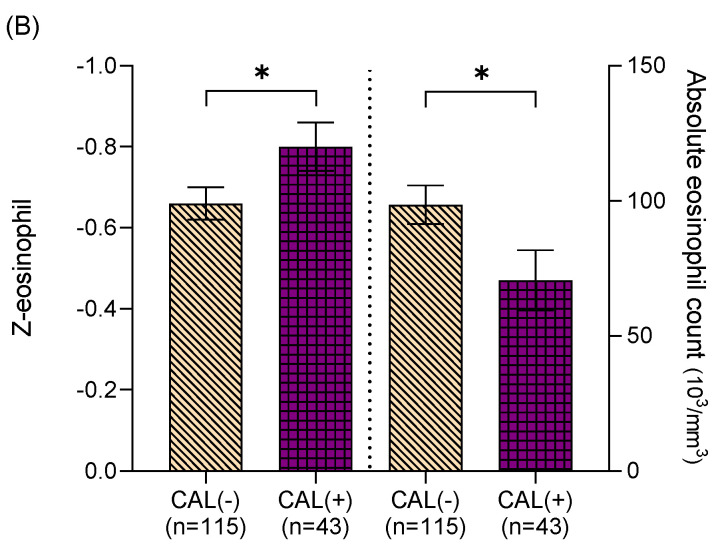
(**A**) Among Kawasaki disease patients with Z-eosinophil ≥ 0, coronary artery lesion (CAL) formation was found in those with higher absolute eosinophil counts (754.92 ± 62.25 cells/mm^3^, vs. 607.91 ± 31.48 cells/mm^3^, *p* < 0.05) and higher Z-eosinophil (3.04 ± 0.17 vs. 2.22 ± 0.19, *p* < 0.05). (**B**) Among Kawasaki disease patients with Z-eosinophil < 0, coronary artery lesion (CAL) formation was found in those with lower absolute eosinophil counts (70.59 ± 11.18 cells/mm^3^ vs. 98.55 ± 7.15 cells/mm^3^, *p* < 0.05) and lower Z-eosinophil (−0.8 ± 0.06 vs. −0.66 ± 0.04, *p* < 0.05). Asterisk (*) indicates a *p* value of < 0.05 between two groups.

We further divided the Z-eosinophil into two groups prior to IVIG treatment. One group was higher eosinophil and above the age baseline (Z ≥ 0, *n* = 240), and the other group was below the age baseline (Z < 0, *n* = 158). In Figure 1A, higher absolute eosinophil counts (754.92 ± 62.25 cells/mm^3^ vs. 607.91 ± 31.48 cells/mm^3^, *p* < 0.05) and Z-eosinophil (3.04 ± 0.17 vs. 2.22 ± 0.19, *p* < 0.05) were found in KD patients with CAL formation in the group of Z ≥ 0. Meanwhile, lower AEC (70.59 ± 11.18 cells/mm^3^ vs. 98.55 ± 7.15 cells/mm^3^, *p* < 0.05) and Z-eosinophil (−0.8 ± 0.06 vs. −0.66 ± 0.04, *p* < 0.05) were noted in KD patients with CAL formation in the group of Z < 0 (Figure 1B). We also analyzed the follow-up echocardiography 2 months post onset of KD, and neither higher eosinophil counts (pre-IVIG) nor Z-eosinophil (pre-IVIG) revealed statistically significant correlations with CAL formation (*p* = 0.99 and *p* = 0.89, respectively).

## 4. Discussion

Since April 2020, multisystem inflammatory syndrome in children (MIS-C) has been described as the combination of severe acute respiratory syndrome coronavirus 2 (SARS-CoV-2)-associated hyperinflammatory response resembling KD and toxic shock syndrome described in children [11]. The pathological hypothesis involves an imbalanced immune activation of both MIS-C and KD, but it seems to have different directions [11,12,13]. In general, respiratory tract virus controls virus replication with type I interferon (IFN) dependent responses in early infection [12]. Unlike common respiratory tract viruses, the SARS-CoV-2 infection can induce a hyperimmune response by suppressing type I and type III IFN responses to delay induction, and therefore, to elevate cytokine storms which result in further inflammation [12,13]. Sacco et al. analyzed serum levels of immune biomarkers and discovered that MIS-C was characterized by prominent T helper type 1 (Th1) with activation of neutrophil and suppressed Th2 response [11]. In addition, the eosinophil-related mediators such as IL-4, IL-5, serum immunoglobulin-E (IgE), and thymus and activation-regulated chemokine (TARC) have been shown to be significantly higher in KD than in age-matched children prior to IVIG treatment and a decrease in IFN-gamma expression has also been reported in KD patients [6,14,15,16]. These results illustrated the tendency to arouse the Th2 response which promoted eosinophil mobilization and the close connection to developing an allergic disease in KD patients. Recently, numerous studies have reported that KD patients had a higher risk for developing allergic diseases [7,17]; however, whether pre-existing allergic diseases lead to a higher risk for KD remains controversial [18,19]. In our study, the prevalence of allergic disease prior to the onset of KD is lower than that of the general population. First, the average age of participants in our study is younger than the peak age of some allergic diseases such as asthma and allergic rhinitis. Second, previous studies have reported a low incidence rate of allergic disease diagnosed before KD [18]. In addition, because these patients did not receive regular childhood treatment at our hospital, a retrospective chart review of data recorded at our hospital may not adequately reflect the prevalence of allergic diseases in our cohort. Therefore, we postulate that the eosinophil count can be a marker for predicting future development of allergic diseases in KD patients. Due to the overlapping clinical features of MIS-C with KD, we searched the literature to investigate the relationship of MIS-C with eosinophil and allergic diseases, and found that eosinophilia or subsequent allergic diseases had not been reported in patients with MIS-C, possibly, because MIS-C tends to have a more prominent Th1 response than a Th2 response, which results in a lower eosinophil count. A longer follow-up time could be required to observe the relationship of MIS-C with allergic diseases.

Normal WBC differential counts according to chronological age are known to differ among age groups [8,9]. We utilized the Z-score of eosinophil counts to adjust for potential age bias. Our study reported that an increase in AEC was found in all age subgroups during the course of KD but statistically significant only in the post-IVIG phase. However, after adjusting with Z-score, the age-adjusted Z-scores of eosinophils all showed significant differences during the pre-IVIG, post-IVIG, and subacute KD courses. Furthermore, we observed that the age subgroup under 1 year old had the most prominent significance of Z-eosinophil in both pre-IVIG and post-IVIG phases, and the age subgroup under 6 months old showed persistently significant differences in the subacute phase. Therefore, whether a patient received treatment or not, a higher incidence of persistent eosinophilia in patients under 6 months old was noted. The findings that younger children have a higher Z-eosinophil as compared with older children may be related to the tendency of a Th2 response in KD and the special feature of immune systems in different age groups [6,16,20]. As compared with adults, newborns have defective Th1-type responses which may make them more vulnerable to infective agents and, thus, enhance the Th2 response and the regulatory T cell (Treg) differentiation [20]. Altogether, the tendency of the neonatal immune system toward Th2 response plus the impact of KD may be the reasons why younger children may have a higher Z-eosinophil. Therefore, the Z-eosinophil seems to be able to predict the development of eosinophil-related allergic diseases during the follow-up of KD, especially among KD patients with an age of onset under 6 months old.

Coronary artery abnormalities are strongly associated with eosinophils [4]. Previous studies have revealed that eosinophilic accumulation begins in the microvessels during the early phase of the histopathology of KD and then, progresses to medium-size muscular arteries, leading to coronary artery lesions [4,21]. However, in our past report, we found that eosinophil count was significantly higher in KD patients without CAL formation after IVIG treatment. We speculated that the phenomenon may be because IVIG decreased the recruitment of eosinophils from local vascular tissues to systemic circulation via transendothelial migration [3,4]. Furthermore, thrombocytosis in KD is also a risk factor for coronary artery complications, and eosinophils have been demonstrated to play important roles in platelet recruitment and activation in the setting of coronary artery lesions [22,23,24]. Our study supported this statement and showed significantly higher platelet counts in the eosinophilia group (*p* < 0.001). Even though the pathogenic mechanism of these observations was poorly defined, these clinical findings indicate some role of eosinophils in the pathogenesis of acute KD vasculitis. Whether eosinophilia is a protective or risk factor for coronary artery lesions during the recovery period after IVIG treatment is still controversial [3,4,25]. Possible factors that can affect the distribution of eosinophils in KD include IVIG product, patient characteristics, aspirin, and other prescribed medications [26,27]. Therefore, we tried to analyze eosinophil only in the acute phase of KD to avoid bias caused by the IVIG product.

In patients with eosinophilia, i.e., a higher eosinophil count than that of average age values, patients have a higher risk of developing coronary artery lesion, while in patients with a lower eosinophil count than that of average age values, patients had a lower risk of developing coronary artery lesion. Defining whether peripheral blood eosinophils have a negative or positive effect on the coronary artery wall during the acute phase of KD is difficult. After reviewing the literature, the anti-inflammation function of eosinophils has been emphasized in recent studies, and this concept is crucial for our results [28]. Two distinct eosinophil subtypes were recently reported with differences in surface protein expression, functions, and response to IL-5, which may explain why eosinophilia has an anti-inflammatory or inflammatory effect in different situations [28,29]. Eosinophils are produced in the bone marrow and released into the peripheral blood, at which point they migrate to inflammatory sites and peripheral tissues for homeostasis [28,29]. Most eosinophils reside in tissues such as thymus, adipose tissue, and lamina propria of the gastrointestinal (GI) tract, and these tissue-resident eosinophils (rEOS) regulate a variety of important biological functions [28]. Recently, lung-resident eosinophils (rEOS), which maturate independently to IL-5, have been found to maintain tissue homeostasis, while inflammatory eosinophils (iEOS), which maturate in an IL-5-dependent manner, were primarily involved in immune responses [28,29]. The eosinophil subtypes are differently involved in asthma pathogenesis [30]. Non-allergic eosinophilic asthma patients had predominant rEOS which prevented Th2-driven airway allergies, while allergic eosinophilic asthma was iEOS predominant, which rapidly infiltrated the airway when allergens attacked [30]. This finding suggests that proportions of eosinophil subtypes can avail in modulating the treatment perspectives for both allergic and non-allergic asthma patients [30]. While no clear explanation for these mechanisms is currently available, we propose that the immune environment may differ in different age groups, thus, affecting the proportions of eosinophil subtypes and having different effects on coronary artery lesions. In the future, further investigation into proportions of eosinophil subtypes may provide more information for allergic disease prediction and specific treatment in children with KD.

Our study has some limitations. First, this study was a retrospectively reviewed study, so some important clinical information may have recall bias from patients and family. Second, the number of patients diagnosed with KD in each age subgroup was small, which may affect the study’s statistical power. Third, this study used a single center, so our results should be examined in another hospital or managed as a cohort study in the future, as eosinophil count has the potential to become an important focus of further research on Kawasaki disease.

## 5. Conclusions

We reported that the Z-eosinophil for the under 6-month-old age subgroup persistently increased significantly as compared with other age groups. We also found that eosinophils may have the opposite function in coronary artery lesions when their count is over or below the average age value, which may be due to different immune environments causing different characteristics of eosinophils. These findings may provide information for clinicians to recognize eosinophil-related allergic diseases during the follow-up of Kawasaki disease, especially in those patients under the age of 6 months old, and eosinophil could be an important predictive factor for allergic disease in KD patients.

## Figures and Tables

**Table 1:** The differences of absolute eosinophil count and Z-eosinophil among age subgroups according to Kawasaki disease patients.

Pre-IVIG Phase (*n* = 398)					
		AEC (cells/mm^3^) Mean ± SE	*p*-value *	Z-Eosinophil Mean ± SE	*p*-value *
<6 mon	(*n* = 36)	514.71 ± 90.37	*p* = 0.076	2.15 ± 0.57	*p <* 0.001
≥6 mon and <1 y	(*n* = 102)	522.07 ± 49.90		2.13 ± 0.33	
≥1 y and <2 y	(*n* = 131)	385.15 ± 31.33		0.65 ± 0.16	
≥2 y and <3 y	(*n* = 59)	463.36 ± 66.21		1.41 ± 0.34	
≥3 y and <4 y	(*n* = 28)	324.18 ± 72.96		0.50 ± 0.35	
≥4 y and <5 y	(*n* = 24)	388.39 ± 69.62		0.52 ± 0.26	
≥5 y	(*n* = 18)	438.86 ± 145.79		0.88 ± 0.68	
**Post-IVIG Phase (*n* = 371)**					
		AEC (cells/mm^3^) Mean ± SE	*p*-value *	Z-Eosinophil Mean ± SE	*p*-value *
<6 mon	(*n* = 34)	444.53 ± 69.77	*p* = 0.004	1.71 ± 0.44	*p <* 0.001
≥6 mon and <1 y	(*n* = 91)	428.97 ± 42.76		1.53 ± 0.28	
≥1 y and <2 y	(*n* = 125)	293.00 ± 23.83		0.19 ± 0.11	
≥2 y and <3 y	(*n* = 55)	354.42 ± 48.42		0.86 ± 0.25	
≥3 y and <4 y	(*n* = 26)	283.13 ± 68.32		0.31 ± 0.63	
≥4 y and <5 y	(*n* = 22)	170.78 ± 27.93		−0.28 ± 0.10	
≥5 y	(*n* = 18)	437.93 ± 266.76		0.80 ± 1.14	
**Subacute Phase (*n* = 236)**					
		AEC (cells/mm^3^) Mean ± SE	*p*-value *	Z-Eosinophil Mean ± SE	*p*-value *
<6 mon	(*n* = 23)	512.38 ± 106.26	*p* = 0.380	2.14 ± 0.67	*p =* 0.004
≥6 mon & < 1 yr	(*n* = 59)	267.39 ± 21.44		0.47 ± 0.14	
≥1 yr & < 2 yr	(*n* = 76)	278.09 ± 22.46		0.12 ± 0.11	
≥2 yr & < 3 yr	(*n* = 40)	389.47 ± 75.64		1.03 ± 0.39	
≥3 yr & < 4 yr	(*n* = 17)	285.99 ± 55.99		0.51 ± 0.29	
≥4 yr & < 5 yr	(*n* = 12)	313.42 ± 83.64		0.25 ± 0.29	
≥5 yr	(*n* = 9)	233.71 ± 54.27		−0.01 ± 0.26	

mon, month; y, year; AEC, absolute eosinophil count; Z-eosinophil, age-adjusted Z-score of eosinophils; IVIG, intravenous immunoglobulin; SE: standard error of mean * Kruskal–Wallis test was used.

**Table 2:** Comparison of age subgroups with the most significant absolute eosinophil counts and Z-eosinophil difference during each Kawasaki disease phase.

**Pre-IVIG Phase (*n* = 398)**			
	<1 year old (*n* = 138) Mean ± SE	1 year old (*n* = 260) Mean ± SE	*p*-value *
AEC (cells/mm^3^)	520.16 ± 43.60	386.69 ± 25.99	*p =* 0.004
Z-eosinophil	2.14 ± 0.28	0.75 ± 0.13	*p <* 0.001
**Post-IVIG Phase (*n* = 371)**			
	<1 year old (*n* = 125) Mean ± SE	1 year old (*n* = 246) Mean ± SE	*p*-value *
AEC (cells/mm^3^)	433.20 ± 36.30	305.36 ± 26.34	*p <* 0.001
Z-eosinophil	1.58 ± 0.24	0.35 ± 0.12	*p <* 0.001
**Subacute Phase (*n* = 236)**			
	<6 months old (*n* = 23) Mean ± SE	6 months old (*n* = 213) Mean ± SE	*p*-value *
AEC (cells/mm^3^)	512.38 ± 106.26	308.05 ± 24.42	*p =* 0.017
Z-eosinophil	2.14 ± 0.67	0.40 ± 0.12	*p =* 0.001

AEC, absolute eosinophil count; Z-eosinophil, age-adjusted Z-score of eosinophils; IVIG, intravenous immunoglobulin.; SE: standard error of mean * Mann–Whitney U test was used.

## Data Availability

Data available on request from the authors.
